# Building with Light: Photoactive Colloids as the Next Generation Meta-Atoms

**DOI:** 10.1021/acs.nanolett.6c00990

**Published:** 2026-04-28

**Authors:** Jinkun Liu, Xiaofeng Li, Yanbin Li, Ruochen Yang, Wei Wang, Jing Zheng, Jingyao Tang

**Affiliations:** † College of Chemistry and Chemical Engineering, 12466 Xiamen University, Xiamen 361005, China; ‡ Department of Chemistry, 25809 The University of Hong Kong, Hong Kong 999077, China; § Materials Innovation Institute for Life Sciences and Energy (MILES), HKU-SIRI, Shenzhen, Guangdong 518055, China; ∥ 66422 Hong Kong International School, Hong Kong 999077, China; ⊥ School of Materials Science and Engineering, 529484 Harbin Institute of Technology (Shenzhen), Shenzhen, Guangdong 518055, China; # HKU-CAS Joint Laboratory on New Materials and Department of Chemistry, Hong Kong 999077, China; ∇ State Key Laboratory of Synthetic Chemistry, The University of Hong Kong, Hong Kong 999077, China

**Keywords:** Photoactive Colloids, Self-Assembly, Optical Materials, Meta-Atoms

## Abstract

At the micro- and nanoscale, active colloids generate mechanical forces that promote aggregation and crystallization. Unlike passive colloids, which assemble at or near thermodynamic equilibrium, active colloids operate inherently far from equilibrium, allowing them to transcend several fundamental constraints of equilibrium systems including detailed balance and reciprocity. Notably, due to the untethered nature and high spatiotemporal precision, light can be employed to modulate the interactions of photoactive colloids with remarkable accuracy and versatility through multiple-mode control, making them promising candidates for next-generation artificial meta-atoms for constructing complex architectures and realizing innovative functionalities. As the material properties of colloidal systems are fundamentally governed by the strength of colloidal interactions, lattice arrangement, and symmetry, the photoactive colloids promised novel active materials with dynamic photonic band gaps, scattering spectra, anisotropy, and effective refractive indices, thereby providing a platform for constructing next-generation optical materials.

## Graphical abstract

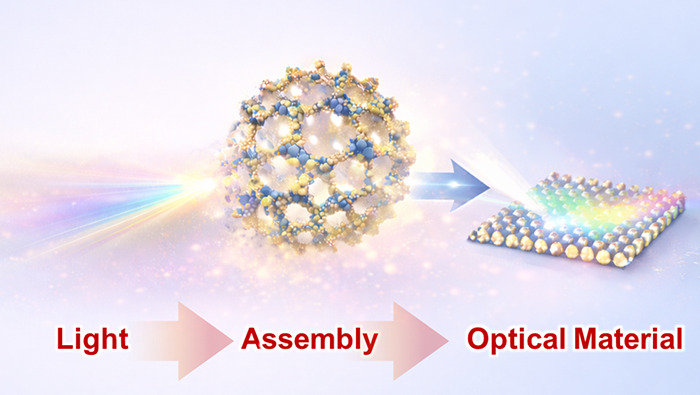

Self-assembly represents one of the most powerful construction principles in nature: from the crystalline formation of opals to the folding of polypeptide chains into functional proteins, complex architectures emerge spontaneously from the bottom-up organization of simple building blocks.^1,2^ In artificial systems, colloids have emerged as particularly attractive assembly units because they provide a unique balance between interaction tunability and dynamics traceability.^3–5^ Their mesoscopic size makes them suitable for real-time manipulation and optical tracking while remaining small enough to preserve thermal fluctuations and reversibility. Consequently, colloids can form long-range ordered structures yet retain the ability to undergo rapid reconfiguration under external fields.^6,7^ Such assemblies can also exhibit metamaterial-like behavior, whereby collective reorganization gives rise to emergent mechanical responses, including soft modes, auxetic deformation, and controllable shape transformation.^8^ In this sense, colloids are not merely enlarged molecules but are better described as artificial meta-atoms, namely, engineered building blocks whose collective organization can generate emergent structural and functional properties beyond those of individual units, positioned between molecular and macroscopic matter. However, conventional colloidal assembly is fundamentally governed by free-energy minimization, where weak interactions, such as electrostatic forces, steric repulsion, hydrogen bonding, or depletion attractions, are delicately balanced against thermal fluctuations on the scale of a few *k*_B_ *T*.^3,9,10^ Consequently, equilibrium assembly is often kinetically slow and, once structures are formed, remains static, making it difficult to achieve reversible reconfiguration and defect self-healing.

The rise of active matter provides a fundamentally different route beyond the equilibrium assembly. Through continuous energy input, structures can be driven, sustained, and reversibly reorganized to a dynamic steady state. Among various active systems, photoactive colloids are particularly compelling: through photophysical effects or photochemical reactions, they induce persistent motion, dissipative assembly, and adaptive self-organization.^4,5^ More importantly, optical actuation inherently offers untethered, high-spatial-resolution, and fully programmable spatiotemporal control, thereby connecting optical fields with the spatial organization of colloidal matter.^3,11,12^ By tuning light intensity,^13^ wavelength,^14^ polarization,^15^ temporal sequences,^16^ or spatial patterns,^17^ photoactive colloidal systems can be driven across multiple organizational states, including dispersed fluid phases,^18^ ordered crystalline structures,^19^ chiral lattices,^20^ and collective swarms.^21^ Additionally, the size of photoactive colloids naturally spans the visible-to-near-infrared range, allowing them to be assembled into innovative optical materials whose properties are determined primarily by their structure rather than their composition. This suggests that optical functions can be directly integrated into the assembly process of photoactive colloids, opening up exciting possibilities for advanced applications in harmonic modulation, intelligent sensing, adaptive camouflage, and dynamic displays.^6,22–24^

Regrettably, the potential of photoactive colloidal materials remains largely untapped, with much of the research still focused on the observation of light-induced aggregation and manipulation and few rudimentary utilizations. This mini-review examines recent advancements in photoactive colloids, emphasizing their potential as building blocks for dynamically reconfigurable optical materials (Figure 1).^22,23,25–31^ We outline the optical modulation methods based on fundamental parameters and categorize the systems by their underlying force-generation mechanisms. Furthermore, we compare how different optical field configurations yield distinct collective dynamics and dissipative assembly pathways, which, in turn, influence structural evolution and tunable optical responses. We hope this review encourages the community to recognize the significant opportunities in translating photoactive colloids into practical and intelligent optical materials.

**Figure 1:**
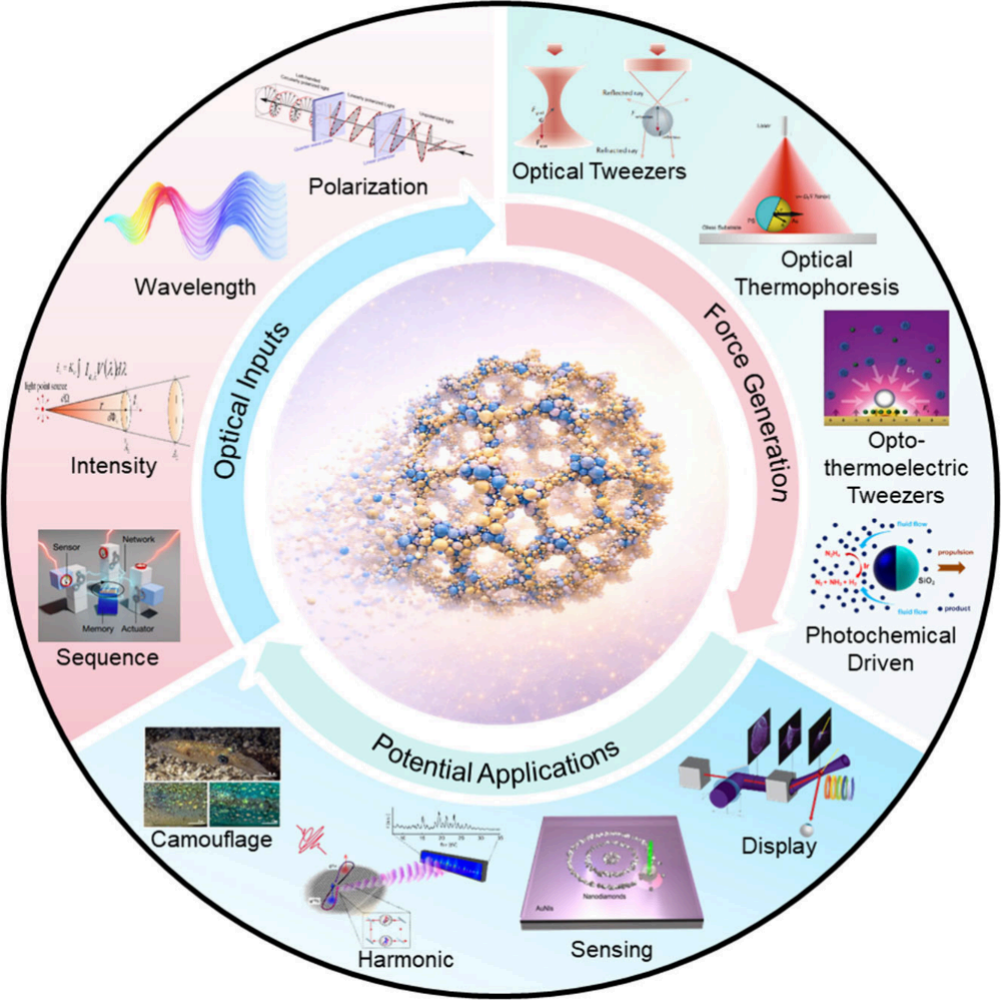
Prospects for photoactive colloids in the engineering of next-generation reconfigurable intelligent materials. Adapted with permission from refs 22, 23, and 25–31 . Copyright^22^ American Association for the Advancement of Science. Copyright^23,29^ American Chemical Society. Copyright^25^ Oxford University Press. Copyright^26–28,31^ Springer Nature.

## Physical and Chemical Basis of Photoactive Colloids

Photoactive colloids generate mechanical effects by harnessing incident photons, a process fundamentally rooted in light-matter interactions. One prominent example of the utilization of light-matter interactions for colloid manipulation is the optical tweezer, where a specially engineered light field is refracted/reflected/absorbed by the nanoparticles, leading to direct/indirect momentum transfer.^32–35^ Optical tweezers are widely recognized as versatile instruments for manipulating objects at the micro- and nanoscale. However, they necessitate intense photon flux because of the extremely low momentum of photons, which can be achieved only with a focused laser. This requirement not only elevates system costs but also imposes fundamental limitations on the scalability of parallel manipulation due to the risks of photodamage and inevitable heating. Photoactive colloids can mitigate this challenge by enhancing the efficiency of optical-to-mechanical energy conversion, thereby enabling optomechanical manipulation under substantially lower illumination intensities.^36,37^ In contrast to the optical tweezers, where the force is generated by the optical pressure,^32–34^ the photon energy is converted into mechanical flow around the photoactive colloids. This is achieved either through physical exploitation of photothermally generated temperature gradients^38,39^ or chemical means involving photochemically generated concentration gradients. Notably, the photochemical manipulation relies on surface photocatalytic reactions to drive asymmetric chemical transformations; these reactions establish local gradients in concentration or electric potential,^40^ thereby inducing motion via diffusiophoresis^17,24^ or electrophoresis.^41,42^ Here, we summarize and compare several main photoactive colloid manipulation techniques and benchmark them with the classical optical tweezers technique, exhibiting distinct trade-offs across driving mechanisms, effective range, force output, and environmental adaptability (Table 1).

**Table 1:** Driving Mechanisms and Comparative Analysis of Manipulation Strategies for Photoactive Colloids

Methods	Driving Mechanisms	Range	Optical Intensity (W/cm^2^)	Force	Adaptability	ref
Optical Tweezers	Gradient Force/Scattering Force	∼25 nm–10 μm	∼10^6^–10^8^	∼0.3 pN	Fuel-Free, High Laser Intensity	^32–34^
Optoelectronic Tweezers	Light-induced Dielectrophoresis/Gradient Force/Scattering Force	∼1 mm	∼1–10^2^	∼1 pN–nN	Fuel-Free, Special Substrate	^43^,^44^
Opto-Thermoelectric Tweezers	Thermoelectric Force/Gradient Force/Scattering Force	∼5–10 μm	∼0.1–10	∼0.4 pN	Special Ionic Surfactant	^31^,^43^,^44^
Opto-Thermophoresis Colloid	Thermo-Osmotic Slip Force	∼100 μm	∼1–10^2^	∼0.2–1 pN	Fuel-Free, Surface Dependent	^38^,^39^,^45^,^46^
Opto-Thermophoresis in CriticalMixture	Local Heat-induced Liquid–Liquid Phase Separation	∼10–500 nm	∼10^–2^–10	∼0.1–10 pN	Critical Mixture solution required	^47–50^
Diffusiophoresis Colloid	Unbalanced Osmotic Pressure	Several Body Length, ∼1–100 μm	∼10^–2^–10	∼0.5–1 pN	Chemical Fuel Required, Environment Sensitive	^17^,^24^
Electrophoresis Colloid	Self-Generated Electrical Force	Several Body Length	∼10^–3^–10	∼0.1 pN-4 nN	Chemical Fuel, Electrical Sensitive	^41^,^42^

### Photophysical Manipulation

The principle of light-driven physical fields involves direct energy dissipation through light-matter interactions, such as photon momentum transfer or photothermal conversion, to create potential fields that disrupt hydrostatic equilibrium. As a paradigmatic example, optical tweezers employ tightly focused laser beams to enable noncontact trapping and manipulation of micro- and nanoscale objects. Mechanistically, the operation of optical tweezers hinges on two fundamental optical forces: the scattering force, which tends to propel objects along the direction of light propagation, and the gradient force, which arises from the spatial inhomogeneity of the optical intensity and draws objects toward the focal point.^51,52^ By employing high numerical aperture objectives to generate steep optical intensity gradients, the axial gradient force is rendered sufficient to overcome the scattering force, thereby forming a stable three-dimensional optical potential well at the focal point (Figure 2a).^33,53,54^ Acting as “tweezers” for the microscopic world, this optical trap enables the precise trapping, stable confinement, and controlled manipulation of atoms, molecules, biological cells, and colloidal particles.

**Figure 2:**
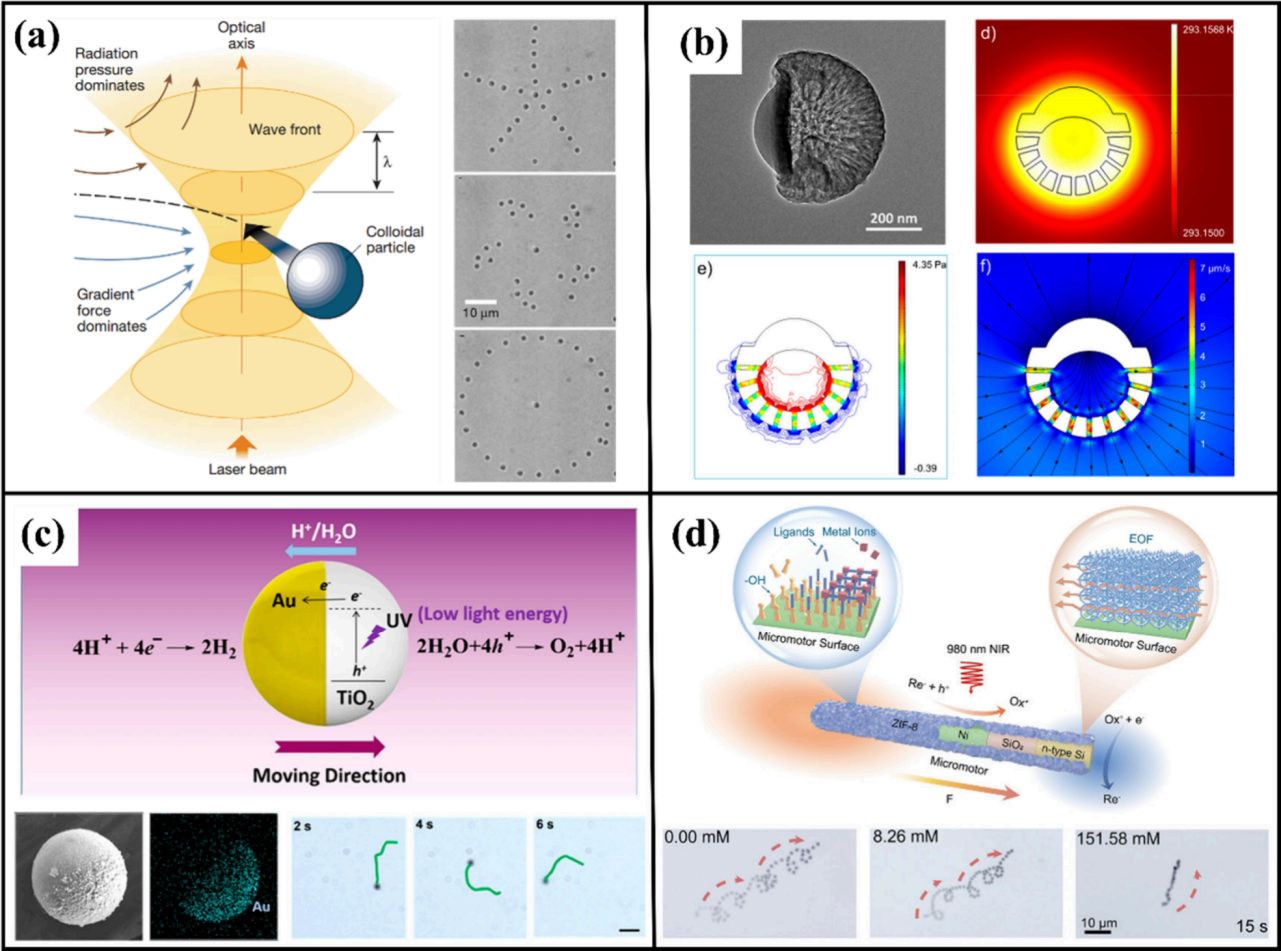
Physical and chemical basis of photoactive colloids. (a) Schematic diagram of optical tweezers utilizing a strongly focused laser beam to create intensity gradients that manipulate and capture active colloidal particles toward the focal point.^54^ (b) Structural design of asymmetric porous hollow carbon particles and simulation of its thermal and flow field distribution under illumination.^55^ (c) Schematic of the self-electrophoresis mechanism in TiO_2_–Au Janus photoactive colloids.^62^ (d) The motion mechanism and actual movement behavior of ZIF-8 porous scaffold photoactive colloids in high-salt physiological environments.^66^ Adapted with permission from refs 54, 55, 62, and 66 . Copyright^54^ Springer Nature. Copyright^55,62^ American Chemical Society. Copyright^66^ WILEY-VCH Verlag GmbH & Co. KGaA, Weinheim.

Conventional optical tweezers are constrained by their narrow operational region and high-power requirements, where several alternative technologies are developed to address these limitations, including optoelectronic tweezers (OET), opto-thermoelectric nanotweezers (OTENT), and thermophoretic active colloids. OET employed structured light and photosensitive substrates to construct “virtual electrode” to induce dielectrophoresis under applied AC electric field,^43,44^ which reduces optical power density by ∼10^5^ relative to traditional optical tweezers, and enabled large-area, scalable manipulation with low-cost optical projection system. OTENT, on the other hand, converts localized photothermal heating with focused laser into electric field via ionic thermoelectric effect without requirement of special substrate or externally applied electric field.^31^ Operating in the microwatt regime, OTENT significantly lowers photodamage risks and overcomes trapping instability in both the Rayleigh and Mie regimes. It enables precise manipulation of particles from 20 to 400 nm, supports diverse materials and geometries, and extends the effective interaction radius to 5–10 μm.

Unlike optical tweezers, which rely on external optical field gradients for actuation, photoinduced self-thermophoresis generates a temperature gradient around the particle through photothermal energy conversion, thereby producing an intrinsic driving force that enables autonomous propulsion. Light-induced self-thermophoretic systems typically leverage the anisotropic architecture of Janus colloidal particles such as dielectric microspheres with hemispherical metal coatings. Upon illumination, the light-absorbing hemisphere (such as gold or carbon layers) generates significant photothermal effects via localized surface plasmon resonance (LSPR) or interband transitions, thereby establishing an asymmetric temperature gradient at the micro/nanoscale (Figure 2b).^45,55,56^

### Photochemical Manipulation

Photochemical propulsion is based on the efficient conversion of optical energy into free chemical free energy. This mechanism typically exploits the photosensitivity of wide–bandgap semiconductors, such as TiO_2_, which generate electron–hole pairs upon photoexcitation. The resulting charge carriers drive asymmetric surface redox reactions—such as the catalytic decomposition of H_2_O_2_ or the hydroquinone/benzoquinone redox cycle—thereby establishing stable, localized chemical gradients at micro- and nanoscale dimensions.^57–59^ The nature of the reaction products determines the propulsion pathway: for neutral molecular species, concentration gradients produce nonelectrolyte self-diffusiophoresis through hydrostatic pressure imbalances near the particle surface, whereas for ionic products with unequal diffusion coefficients, charge separation gives rise to electrolyte self-diffusiophoresis.^60,61^ On the other hand, if ions are asymmetrically generated on different part of a colloidal particle, an electric field is generated across the particle surface, leading to self-electrophoresis.^13,62,63^ In particular, self-electrophoresis is advantageous for micro- and nanoscale manipulation because its electrically driven mechanism enables circuit-like integration and control, allowing currents to be routed and modulated in a programmable manner both internally and externally.^64,65^ A common strategy to realize this mechanism is to introduce structural asymmetry into Janus architectures. A representative example is the TiO_2_–Au Janus particle, which achieves self-electrophoretic propulsion under UV illumination.^62^ Here, photoinduced charge separation induces asymmetric ion fluxes, creating a self-generated electric field that powers efficient motion with precise optical switchability (Figure 2c). Beyond structural design, material composition critically influences propulsion by modulating catalytic activity and interfacial potential differences, which control asymmetric ion fluxes and the resulting self-generated electric field.^63^

However, photoactive colloids relying on ionic propulsion mechanisms, including self-electrophoresis and electrolytic self-diffusiophoresis, face significant challenges in physiological high-salt environments, where ionic screening markedly weakens their propulsion velocity. In particular, self-electrophoretic colloids are especially susceptible to this effect because compression of the electrical double layer severely impairs the self-generated electric field required for propulsion.^3,4,66–68^ The ion tolerance of any given active colloid can be benchmarked by EI_50_, which is defined as the electrolyte concentration at which the colloid velocity decreases to 50% of its initial value.^68^ In most self-electrophorectic colloid, the EI_50_ is below 1 mM, way below the physiological media (∼140 mM). With careful surface electrokinetic analysis, it was shown that the electrophoretic force generation is highly dependent on the surface state of particles, where the polymeric coating and porous structure can help overcome the electrical double layer compression, allowing enhanced ion tolerance. Polymeric coatings—such as polyelectrolyte films^68^ or amino-acid layers^67^—enhance surface conductivity and preserve effective electrokinetic coupling, thereby alleviating the collapse of the electrical double layer; such modifications typically increase EI_50_ by more than 2 orders of magnitude (often exceeding ∼100–200×), enabling reliable operation in physiological buffers.^69^ In addition, polymeric encapsulation strategies have demonstrated the capability of light-driven micromotors to operate in complex biological media, including serum and blood samples, highlighting their practical biocompatibility.^69^ In contrast, porous structure^66,70^ (for example, MOF shells shown in Figure 2d) introduce ion-permeable pathways that decouple propulsion from the interfacial Debye layer, allowing charge transport through the particle body rather than being confined to the surface. This mechanism can elevate EI_50_ by over ∼200×, and sustain stable motion at blood-level ionic strength.

### Light-Induced Assembly of Photoactive Colloids

As a remotely programmable “architect”, light dynamically tunes interparticle forces, assembling photoactive colloids into reconfigurable nonequilibrium structures. Unlike passive self-assembly, these colloids remain persistently out of equilibrium due to continuous energy influx, which breaks the detailed-balance and enables nonreciprocal interactions.^6,71,72^ These active interactions enable dissipative systems to assemble not only faster, but also access more complex phases,^73^ where dynamic patterns such as collective swarms,^17,74^ chiral crystallites,^20^ and motility-induced phase separations^6^ can emerge. These emergent patterns and dynamic structures can be interactively controlled using light, achieving a high spatiotemporal response for the desired functions. From the perspective of building blocks, the assembled structure can be constructed from single- or multiple-component colloids, allowing interactions between different colloid species to be modulated independently to construct desired structures, ranging from single-component superlattices to colloid compounds (Figure 3).

**Figure 3:**
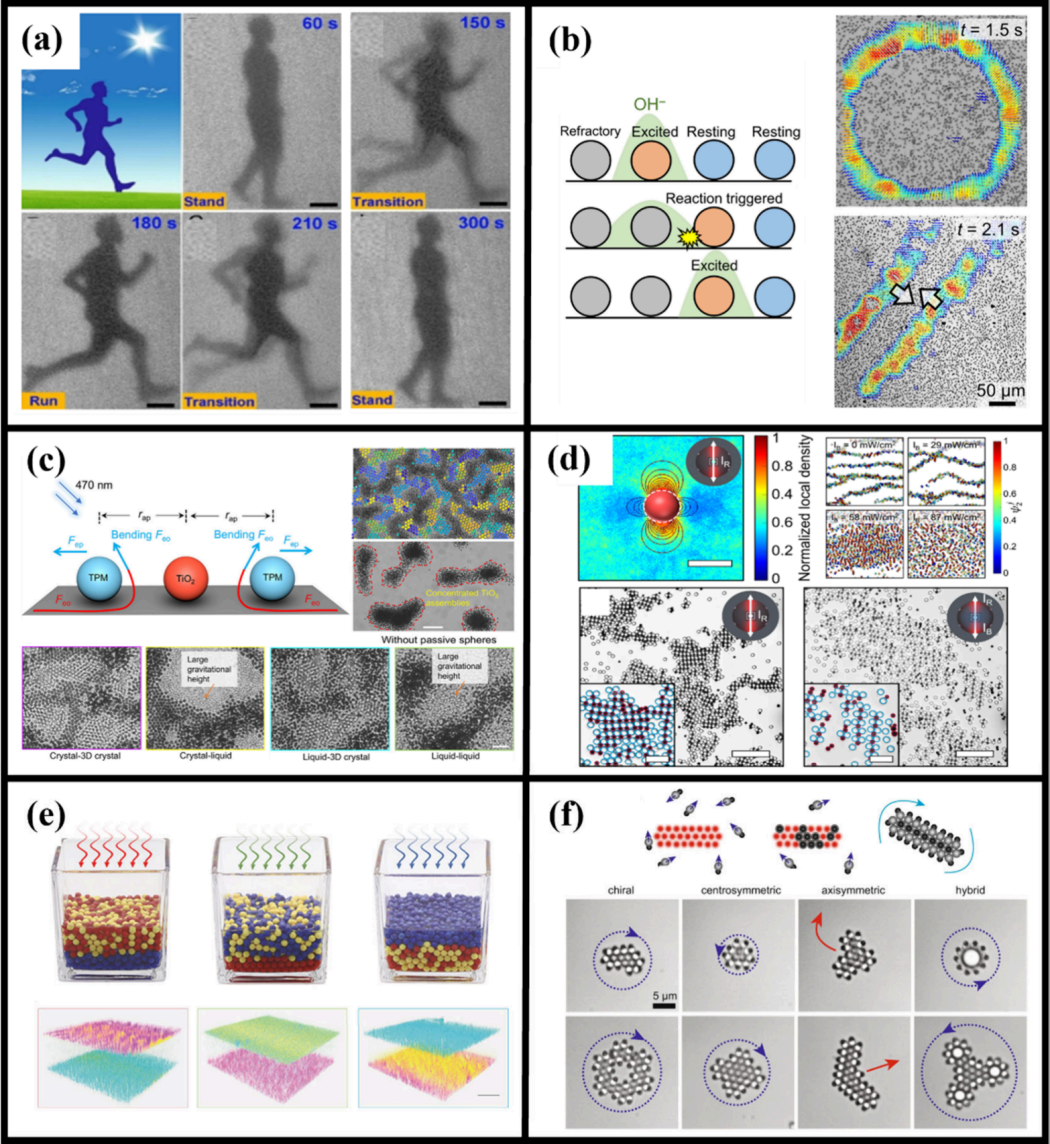
Light-induced assembly of photoactive colloids. (a) A light-driven molybdenum disulfide colloidal particle collective system enables programmable dynamic assembly of colloidal particle clusters.^17^ (b) Photoactive colloids exhibit periodic, wave-like motion synchronized by traveling OH^–^ chemical waves that trigger sequential Ag/AgCl oscillatory reactions under UV light.^74^ (c) Photoactive particles drive the dissipative assembly and reversible reconfiguration of passive colloids through light-induced dynamic balance.^83^ (d) Evolution of photoactive colloidal swarms from meta-atom assembly to chemical communication-driven regulation.^7^ (e) Wavelength-selective photoactive colloidal cluster phase separation enables multicomponent phase separation and structurally programmable reconfiguration.^6^ (f) Photoactive colloid-driven self-assembly of passive component lattices.^85^ Adapted with permission from refs 6, 7, 17, 74, 83, and 85 . Copyright^6,85^ Springer Nature. Copyright^7,83^ American Chemical Society. Copyright^17^ WILEY-VCH Verlag GmbH & Co. KGaA, Weinheim. Copyright^74^ American Association for the Advancement of Science.

### Single-Component Photoactive Colloids Assembly

The assembly of single-component photoactive colloids may be conceptually referred to as the equivalent of a construction elementary substance in chemistry. In these systems, identical particles convert incident light into local chemical, thermal, or electric gradients, which in turn generate effective interactions via diffusiophoretic attraction, thermophoretic coupling, or hydrodynamic flows.^12,17^ Unlike equilibrium self-assembly driven by free-energy minimization, active interactions can be customized in terms of strength, range, and symmetry. This adaptability makes the transformation of colloid allotropes more prevalent and straightforward. Consequently, active colloid assemblies can be reorganized and disassembled reversibly in real time, offering an unprecedented structural plasticity.

For geometrically symmetric particles subjected to uniform illumination, the resulting active interaction typically reflects the symmetry of the colloid composition.^75^ In the case of the most commonly used spherical colloids, the induced field tends to be omnidirectional, often resulting in close-packed active aggregation or crystalline structures.^76^ Conversely, spontaneous symmetry breaking, nonlinear many-body coupling, and the feedback between particle motion and the fields they generate can give rise to the emergence of exotic collective phases,^77,78^ which are the focus of ongoing research in the field of active matter, although they are not the primary focus of this article.

Structured light fields further expand the design space of single-component assemblies by providing high-dimensional spatiotemporal control. Spatial modulation of illumination can create programmable interaction landscapes that guide particles into predefined patterns or reconfigurable architectures. For example, spatial modulation of light-intensity gradients can induce swarms of molybdenum disulfide (MoS_2_) microparticles to form dynamically reconfigurable assemblies capable of reversible transformations (Figure 3a).^17^ Furthermore, by utilizing the photonic nanojet effect, originally symmetric spherical titanium dioxide (TiO_2_) particles can form localized reaction centers on their shadowed sides; this induced high-resolution anisotropic interaction allows the system to achieve on-demand switching between all five two-dimensional Bravais lattices within a single-component system.^24^ This highly reversible and flexible dynamic assembly strategy overcomes the structural rigidity inherent in conventional passive assembly.

In addition to spatial order, photoactive colloids with autocatalytic reactions may give chemical oscillations with spatial and temporal synchronization. For example, silver (Ag) half-coated Janus microspheres spontaneously oscillate due to oscillatory chemical reactions.^21,74^ These oscillating colloids act as chemically coupled oscillators, and when approaching one another, spontaneously synchronize their pulsing frequencies and phases through the exchange of chemical signals, forming pulsating clusters characterized by periodic contraction and expansion (Figure 3b).^74^ As the population density further increases, this local synchronization triggers long-range reaction-diffusion waves (such as OH^–^ chemical waves), driving discrete Ag Janus particles to migrate orderly in the form of “ballistic waves” or “swarming waves”, thereby achieving long-range, distortion-free information relay without external intervention.^21,74,79,80^ The dynamics and spatial features of such waves can be further modulated by structured light.^81^

### Assembly of Multicomponent Photoactive Colloids

The interaction of multiple photoactive colloids added additional complexity and design space to material construction, which may be referred to as the “chemical reaction” or mixing of colloidal reactants.^82^ Unlike single-component systems, where clustering arises primarily from self-generated fields and many-body coupling among identical particles, multicomponent mixtures inherently break interaction symmetry through species-dependent activity, mobility, or responsiveness. This asymmetry gives rise to nonreciprocal couplings and directional interactions that cannot be realized in homogeneous systems, enabling assembly governed primarily by activity heterogeneity.^19,83–85^

In mixtures where a minority of active particles is dispersed within a passive background, the active species can function as dynamic organizers that mediate interactions among otherwise weakly interacting particles.^19^ Even a minute fraction of photoactive colloids can act as nucleation centers, recruiting large numbers of passive particles through photocatalytically generated chemical gradients that produce long-range diffusiophoretic attraction, thereby forming finite clusters or extended crystalline assemblies. The resulting assemblies arise from a balance between long-range attraction and short-range repulsion produced by competing phoretic, electro-osmotic, and electrophoretic flows, stabilizing ordered binary phases with characteristic interparticle spacing that differs fundamentally from equilibrium crystals (Figure 3c).^83^ Furthermore, modulation of optical parameters can alter propulsion direction and surface reaction asymmetry, switching effective interactions between attraction and repulsion and enabling reversible fusion, fission, and reconfiguration of clusters.^86^

When multiple active components are present, clustering behavior evolves into collective organization governed by symmetry breaking, activity contrast, and nonreciprocal coupling between species. Differences in propulsion mechanisms, optical response, or interaction range generate complex interaction networks that support higher-order structures such as chains, bands, or ordered active crystals, and mixtures of distinct active particles can form composite clusters with well-defined stoichiometry, effectively mimicking chemical reactions or alloy formation at the mesoscale (Figure 3d).^7^ Spectral selectivity further enhances programmability: by encoding particles with distinct absorption bands, individual components can be selectively activated by specific wavelengths, leading to wavelength-dependent segregation, layered organization, or cooperative phase behavior (Figure 3e).^6^ The interplay of nonreciprocal forces and dynamic feedback can also produce functional architectures, including autonomous micromachines assembled via optical templating, whose motion arises from internal force generation and geometry (Figure 3f),^85^ as well as dissipative photonic states emerging from density-dependent clustering, such as dynamically tunable random lasing controlled by the instantaneous structure of the assembly.^84^

## Applications and Function Demonstrations

Benefiting from programmable geometries, physical morphologies, and assembly modes, active colloidal particles are envisioned as ideal “meta-atoms” for the bottom-up construction of functional devices. Via versatile manipulation strategies, these particles can be orchestrated into programmed macroscopic architectures, eliciting collective synergies that surpass the capabilities of individual constituents. This section highlights the burgeoning applications of photoactive colloids, systematically examining their seminal contributions to micromanipulation and delivery, micronanophotonics (e.g., random lasers, photochromic displays, and chiral optical manipulation), and high-efficiency catalysis.

### Micromanipulation and Delivery

Photoactive colloids, functioning as micronanorobotic platforms, have demonstrated distinctive superiorities in precise spatiotemporal manipulation and on-demand cargo delivery across micronanoscale regimes. Leveraging the superior spatiotemporal controllability of light fields, these colloids can be precisely guided to function as “micro-vehicles”, facilitating the capture, loading, long-range transport, and site-specific release of microspheres, living cells, and drugs.^87,88^ Recent studies have demonstrated the intelligent transport and precise manipulation of diverse passive cargoes using TiO_2_ active colloidal swarms, enabled by the synergistic integration of three distinct kinetic mechanisms: photothermal convection, diffusiophoresis, and electrophoresis (Figure 4a).^88^ This platform facilitates sophisticated collaborative behaviors that transcend the capabilities of individual active units, allowing the swarms to autonomously capture and migrate heavy payloads over long distances. By leveraging the height-dependent velocity profile inherent to microfluidic channels, the system achieves spontaneous size-based cargo sorting. Furthermore, through the precise modulation of incident light intensity, the swarms can execute sequential hierarchical cargo release and repetitive cyclic delivery protocols.

**Figure 4:**
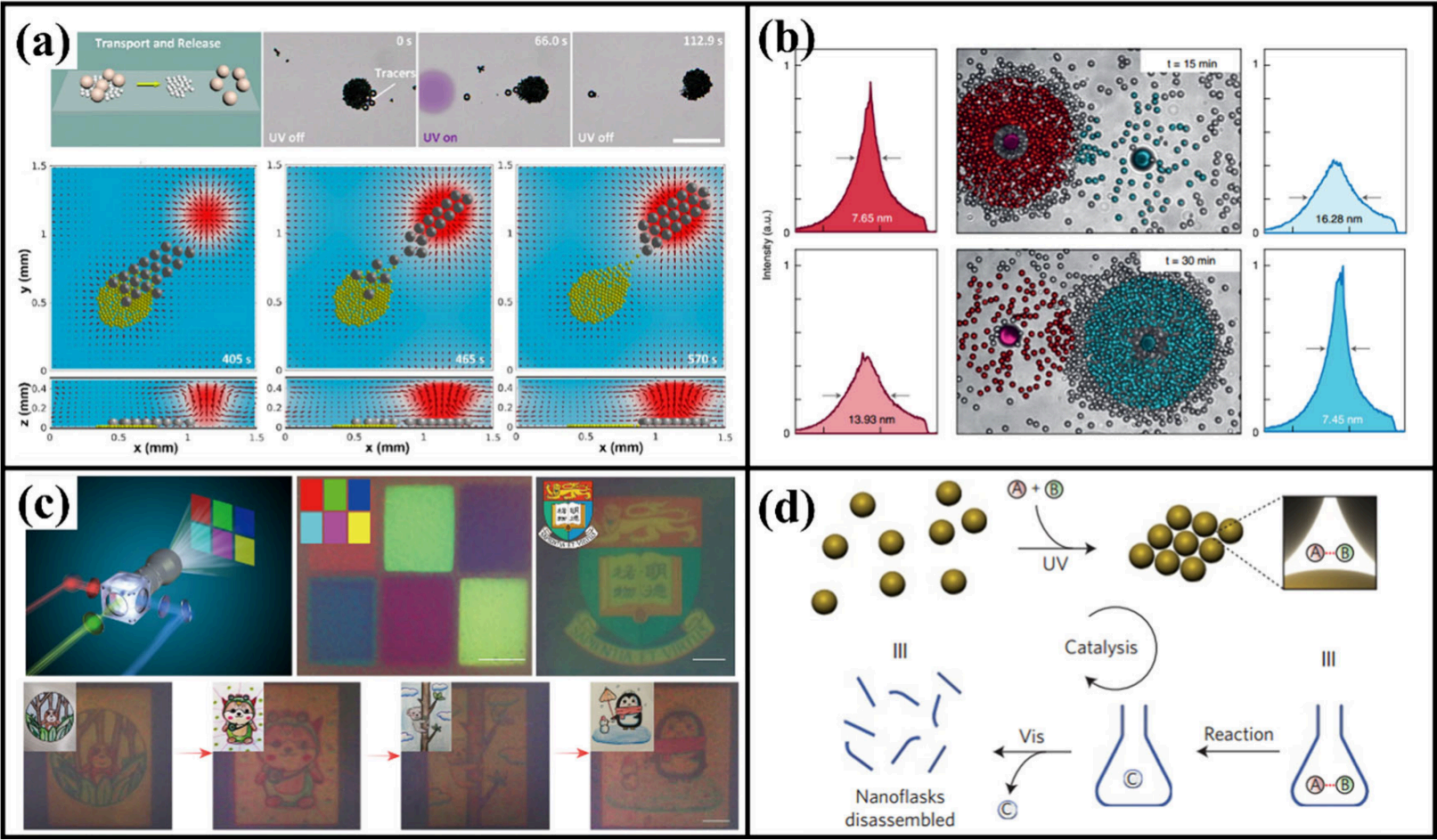
Applications and function demonstrations. (a) Intelligent transport and cargo sorting of light-driven TiO_2_ active swarms.^88^ (b) Photoactive colloids as reconfigurable scattering media for high-performance random lasing.^84^ (c) High-resolution photochromic displays via wavelength-dependent photoactive colloidal phase separation.^6^ (d) Photoactive colloidal reconfigurable platforms for sustainable catalysis.^92^ Adapted with permission from refs 6, 84, 88, and 92 . Copyright^6,84,92^ Springer Nature. Copyright^88^ American Chemical Society.

### Micronanophotonics

Because the size of colloids is inherently comparable to optical wavelengths, their spatial organization directly governs key optical properties such as scattering, interference, and effective refractive index, thereby establishing a powerful platform for micronanophotonic systems. This section highlights their applications in random lasers, photochromic displays, and chiral optical manipulation.

A random laser is a special light source that does not rely on an external resonant cavity but instead utilizes multiple scatterings within a disordered medium to achieve optical amplification. In active colloidal systems, randomly distributed colloidal particles (such as rutile TiO_2_ or plasmonic micro/nanomaterials with broadband scattering capabilities) can act as strong scatterers; when the pumping laser energy reaches a threshold, the emission spectrum narrows significantly and its intensity surges.^89^ Photoactive colloids provide a new approach to addressing the challenge of real-time reconfiguration in random lasers: the distribution of particles can be dynamically manipulated through a dual-beam system (one beam for inducing colloidal aggregation and the other for pumping). When the inducing laser is turned off, the assembly disperses and the lasing action ceases; by reaggregating particles through a dissipative assembly process, repeatable laser emission with highly consistent spectral characteristics can be achieved (Figure 4b).^84^ Recently, researchers have employed holographic optical tweezers to precisely construct colloidal clusters in three dimensions, which not only initiates random lasing but also lays the foundation for developing parallel laser arrays and networked random lasers.^90^

In photochromic color displays, photoactive colloids can dynamically adjust their assembly behavior based on the wavelength of incident light, enabling high-resolution color patterns (Figure 4c).^6^ Differences in light absorption among colloids induce selective interparticle attraction and wavelength-dependent separation. For instance, projecting a polychromatic beam of red, green, blue, cyan, magenta, and yellow light onto a colloidal mixture generates precise color patterns in designated areas within seconds. Thanks to their programmable and reconfigurable nature, these phase-separation patterns can be erased and regenerated repeatedly without a loss of quality.

Chirality, a geometric property where structures are nonsuperimposable on their mirror images, can be emulated in active colloidal systems through biomimetic design. Opto-thermoelectric tweezers generate light-driven local temperature gradients, creating thermoelectric fields that, together with micelle-mediated dissipative forces, enable “atom-by-atom” assembly of colloidal particles such as gold, silicon, or polystyrene into precise chiral configurations. This fully optical approach allows in situ disassembly and reconfiguration into enantiomers or other isomers, providing dynamic, on-demand control of chiroptical properties.^20^

### High-Efficiency Catalysis

Catalysis plays a pivotal role in modern industry, with approximately 85% of manufactured goods relying on catalytic processes across strategic sectors including healthcare, energy, materials science, and electronics. To address the escalating demand for high-efficiency production, photoactive colloidal assembly has emerged as a transformative approach. By precisely engineering catalytically active materials at the micro/nanoscale and creating unique confined reaction environments, this technology significantly enhances catalytic performance. These systems optimize reaction pathways through the integration of catalytic components or the formation of internal “nanoflasks” within the assemblies. For example, visible light can trigger the coassembly of CdSe/CdS quantum dots with platinum nanoparticles via surface ligand oxidation.^91^ This process not only increases local nanoparticle density but also facilitates synergistic electron transfer between the photocatalyst and cocatalyst, markedly improving the efficiency of photocatalytic water splitting. Furthermore, UV-induced aggregation of azobenzene-functionalized gold nanoparticles creates micronano interstices that act as confined reaction volumes (Figure 4d).^92^ These spaces effectively enrich reactants and modulate the reaction kinetics, thereby promoting the dimerization of anthracene derivatives and the hydrolysis of acetals. Leveraging the dynamic reconfiguration of photoactive swarms under alternating UV–visible irradiation, we can determine these particles can undergo multiple reversible cycles of assembly and disassembly. Such adaptivity provides the catalytic system with exceptional structural flexibility and reconfigurability, laying the groundwork for complex sequential reactions and the development of intelligent, sustainable chemical processing platforms.

Despite these encouraging advances, photoactive colloids still require further improvements in propulsion robustness, control precision, and environmental adaptability to support broader practical applications. More generally, continued progress in responsive material design, external field programmability, and system level integration will be essential for moving photoactive colloids from proof-of-concept studies toward practical technologies.

## Prospects of Photoactive Colloids for Optical Materials

Compared with traditional optical materials, photoactive colloids utilize light as a remote, programmable “architect” to guide colloidal assembly, creating a novel paradigm for bottom-up material design. This approach transforms matter from static, fixed structures into dynamic, reconfigurable systems, endowing optical materials with enhanced flexibility and controllability. Owing to its unique advantages—including noncontact actuation, ultrahigh spatiotemporal resolution, wavelength selectivity, and polarization control—light enables independent addressing of different regions within a material, or even individual particles. By controlling the lattice symmetry and lattice constant during assembly, optical fields can effectively “write” the photonic band structure and its response directly into the material. Because the properties of photonic crystals fundamentally arise from periodic modulation of the refractive index, even minor structural variations can induce pronounced spectral shifts or phase transitions. Consequently, light-driven assembly provides a powerful route to achieving dynamically programmable optical functionalities.

Importantly, light-driven assembly opens new opportunities for realizing low-density, open lattices that are difficult to obtain through equilibrium self-assembly. Traditional colloidal crystallization typically favors densely packed structures^93,94^ (e.g., hexagonal close-packed (HCP) and face-centered cubic (FCC) structures) because these minimize free energy. However, many desirable photonic architectures—such as kagome and diamond lattices^95,96^ —require low coordination numbers and directional interactions. These structures can support complete photonic bandgaps and unusual dispersion relations but are notoriously difficult to fabricate using conventional approaches, often requiring highly anisotropic “patchy” particles, DNA-programmed interactions,^95^ or complex templating strategies.^97^ Light-driven assembly offers a fundamentally different route: nonequilibrium interactions sustained by continuous energy input can stabilize thermodynamically unfavorable configurations, while active propulsion helps the system escape kinetic traps and guides particles toward low-filling-fraction lattices with open band structures.^98^ Thus, light not only initiates assembly but also supplies the energy necessary to maintain these open architectures.

Beyond structural construction, optical control enables functionalities that are unattainable in traditional colloidal materials, including on-demand switching between lattice types, adaptive defect healing, spatial patterning of heterogeneous structures, and dynamic responses synchronized with external signals.^3^ These capabilities point toward a vision of programmable photonic matter in which materials behave less like static objects and more like field-controlled information processing systems. Within this framework, each colloidal particle can be regarded as an energy-transducing “meta-atom,” whose interactions are defined not only by intrinsic material properties but also by the applied optical field in real time.

Despite their great promise, translating photoactive materials into practical applications still faces a series of fundamental challenges. The central issue is how to use light to sculpt particle behavior across multiple scales, enabling precise control over interparticle interactions, independent addressing of different regions within a material—or even individual particles—and the effective conversion of local responses into collective optical functionality. Achieving this goal requires integrating photochemical driving with molecular recognition strategies to construct active lattices that combine structural precision with reversibility. At the same time, inverse design approaches are needed to map desired optical functions onto dynamically evolving many-body systems.^99,100^ Because active particles exhibit strong nonlinear coupling and illumination defines a high-dimensional control space, conventional trial-and-error methods are largely inadequate. Artificial intelligence models capable of learning structure–response relationships are therefore expected to become key tools for exploring the design space of nonequilibrium photonic matter and enabling predictive programmability.

In summary, photoactive colloids establish a fundamentally new paradigm for materials construction by functioning as light-powered, reconfigurable meta-atoms capable of converting photon energy into programmable mechanical interactions and collective organization. Unlike conventional static building blocks, these active units operate far from equilibrium, enabling the reversible assembly, restructuring, and adaptation of architectures across multiple length scales under spatiotemporally controlled illumination. Through precise regulation of particle behavior and interparticle interactions, photoactive colloids make it possible to dynamically “write” structure and functionality into matter, opening unprecedented opportunities for adaptive photonic materials, intelligent microsystems, and responsive metamaterials. Although challenges remain in achieving robust control in complex environments, scalability, and integration into practical devices, continued advances in materials design, structured light engineering, and theoretical understanding are expected to transform these systems from laboratory demonstrations into versatile platforms for next-generation active materials.
